# Molecular cloning and expression analysis of two key genes, HDS and HDR, in the MEP pathway in *Pyropia haitanensis*

**DOI:** 10.1038/s41598-017-17521-9

**Published:** 2017-12-13

**Authors:** Yuan He, Zhihong Yan, Yu Du, Yafeng Ma, Songdong Shen

**Affiliations:** 1 Department of cell Biology, School of Biology and Basic Medical, Soochow University, No. 199 Renai Road, Suzhou, China; 2 Aquaculture technology extending station of Xiuyu District, Putian, China

## Abstract

The 1-hydroxy-2-methyl-2-(*E*)-butenyl-4-diphosphate synthase (HDS) gene and the 1-hydroxy-2-methyl-2-(*E*)-butenyl-4-diphosphate reductase (HDR) gene are two important genes in the 2-C-methyl-D-erythritol 4-phosphate (MEP) pathway. In this study, we reported the isolation and characterization of full-length HDS (MF101802) and HDR (MF159558) from *Pyropia haitanensis*. Characteristics of 3-D structures of the *PhHDS* and *PhHDR* proteins were analysed respectively. The results showed that the full-length cDNA of *PhHDS*, which is 1801 bp long, contained a 1455 bp open reading frame (ORF) encoding a putative 484 amino acid residue protein with a predicted molecular mass of 51.60 kDa. Meanwhile, the full-length cDNA of *PhHDR* was 1668 bp and contained a 1434 bp ORF encoding a putative 477 amino acid 2 residue protein with a predicted molecular mass of 51.49 kDa. The expression levels of the two genes were higher in conchocelis than that in leafy thallus. Additionally, the expression levels could be influenced by light, temperature and salinity and induced by methyl jasmonate (MJ) and salicylic acid (SA). This study contributed to our in-depth understanding of the roles of *PhHDS* and *PhHDR* in terpenoid biosynthesis in *Pyropia haitanensis* and the regulation of the two genes by external environments.

## Introduction

*Pyropia haitanensis* (Bangiales, Rhodophyta) is one of the most important economic algae in China and has great value in research^1^. This alga is distributed in the intertidal zones from the temperate rocky shores and has a biphasic life cycle including gametophytic blade and filamentous conchocelis phases^2,3^. As a typical warm temperate zone species, it is cultivated along the coast of the South China Sea^4^, and its output accounts for 75%-80% of the total production of *Pyropia* in China every year^5^. Because of tidal effects, light, temperature, salinity and other environmental factors that may result in cyclical changes in intertidal zone, *Pyropia haitanensis* has a unique physiological adaptation mechanism. When the tide comes, the algae is exposed to air for up to 4 h or more, and its water loss rate reaches up to 90% or more, which reveals its strong resistance to the environment. Therefore, *Pyropia haitanensis* is regarded as an ideal material for studying adaptability to complex intertidal zone environments^6^.

**Figure 1:**
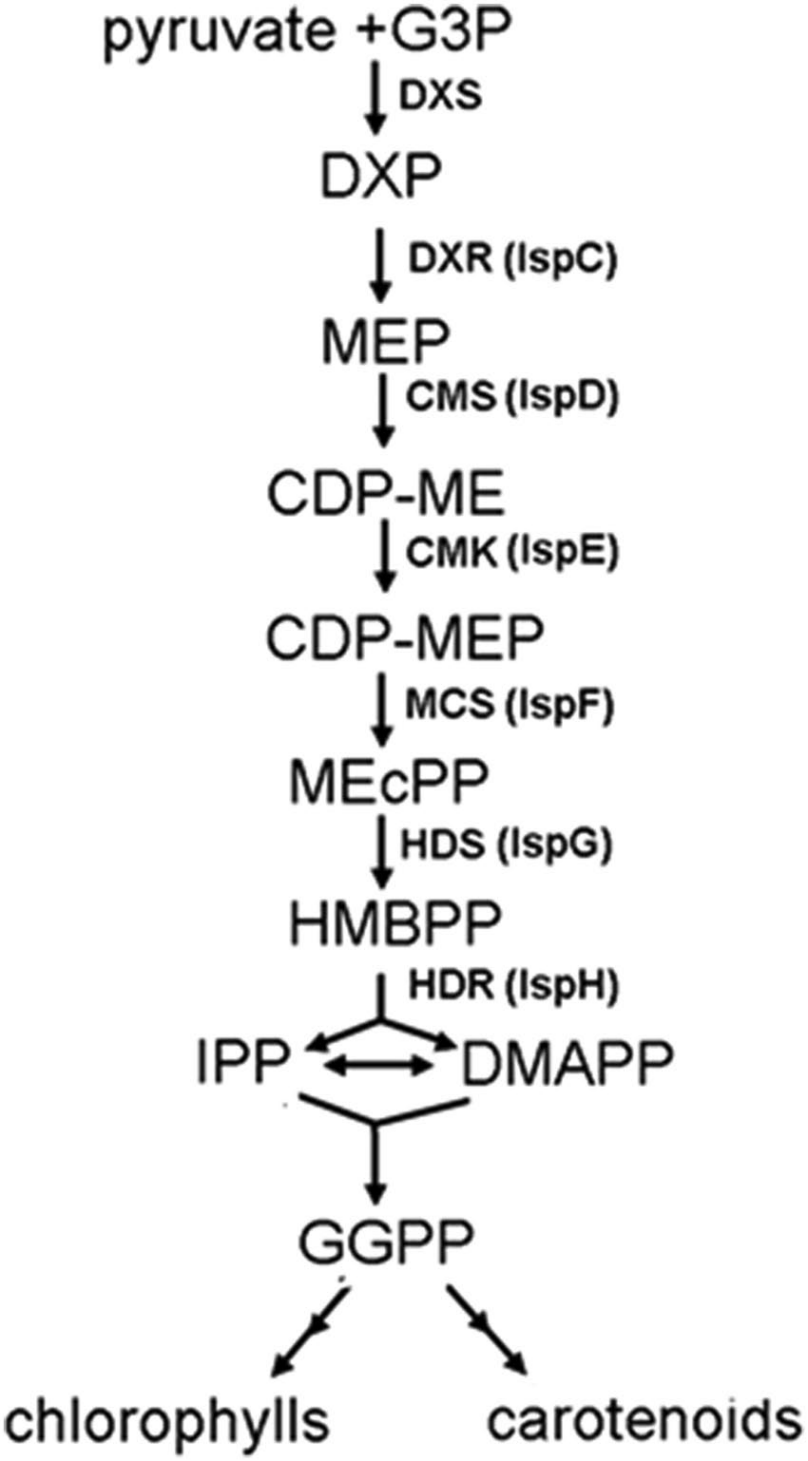
The MEP pathway for isoprenoid biosynthesis in plants. Seven enzymes participate in the MEP pathway. HDS and HDR are the enzymes that regulate the last two steps in terpenoid metabolism in the MEP pathway. Isopentenyl pyrophosphate (IPP) as a precursor for the downstream terpene biosynthesis and the difference between the two is formation mechanisms of IPP and its isomer diallylene pyrophosphate (DMAPP). Geranyhlgeranyl diphosphate (GGPP) is produced by plastidic GGPP synthase (GGPS) and serves as a precursor for metabolic branches, including chlorophylls, carotenoids.

Terpenoids are a kind of biological secondary metabolite that are widespread in nature. The metabolic product is composed of isoprene as a unit. There are more than 23000 different isoprenoids in nature, and half of them are found in plants^7^. Terpenoids are synthesized by two biological pathways: the MVA pathway and the MEP pathway. The MEP pathway is not dependent on mevalonic acid (MVA) for the IPP synthesis. It is known that the deoxyxylulose-5-phosphate pathway (DXP) or methylerythritol 4-phosphate pathway (MEP) is an important way to synthesize terpene compounds (Fig. 1). The MEP pathway exists in pathogens, algae and higher plants, including important human pathogens, such as Mycobacterium tuberculosis and malaria parasites^8,9^. The two pathways both use isopentenyl pyrophosphate (IPP) as a precursor for downstream terpene biosynthesis, and the differences between the two pathways are the formation mechanisms for IPP and its isomer diallylene pyrophosphate (DMAPP). Moreover, the MEP pathway exists in plastids, and 1-xyloxyl-5-phosphate (DXP) is its key precursor material^10^.

The 1-hydroxy-2-methyl-2-(*E*)-butenyl-4-diphosphate synthase (HDS) and 1-hydroxy-2-methyl-2-(*E*)-butenyl-4-diphosphate reductase (HDR) genes participate in the last two steps of terpenoid metabolism in the MEP pathway. HDR is a key enzyme in the MEP pathway^11^. HDS has been fully characterized in *Solanum lycopersicum*, *Hevea brasiliensis*, *Arabidopsis thaliana* and *Ginkgo biloba* ^12–15^. HDS is considered a potential regulator of carbon flux in the MEP pathway; HDS also participates in plant defence mechanisms^16^. HDR simultaneously synthesizes IPP and DMAPP in the last step of the MEP pathway^17^ and acts as a restricting factor for isoprenoid biosynthesis in *Escherichia coli* ^18^. HDR has also been cloned and analysed from many species, such as *Hevea brasiliensis*, *Arabidopsis thaliana*, *Ipomoea batatas* and *Camptotheca acuminata* ^19–22^. However, neither the HDS nor HDR genes have been cloned and analysed from *Pyropia*.

Our research group aimed to explore the metabolic status of the MEP pathway in *Pyropia haitanensis* by studying the expression of *PhHDS* and *PhHDR* under different environmental stresses and elicitor treatments. In this study, we obtained the full-length cDNA sequence of the two genes, which are the last two steps of the terpenoid metabolism in the MEP pathway, and we analysed the putative proteins encoded by the two genes using bioinformatics analysis. The results helped further our understanding of these two important genes in the MEP pathway and provided a practical way to study the molecular mechanism associated with terpenoid biosynthesis in *Pyropia haitanensis*. Moreover, analysis of the expression levels of these terpenoid biosynthesis genes in different environments will prove that *Pyropia haitanensis* has a unique physiological adaptation mechanism to different environments.

## Results

### Cloning the full-length cDNA of *PhHDS* and *PhHDR*

The primers for cloning *PhHDS* (HDSF/HDSR) were designed based on the conserved region in HDS genes from *pyropia yezoensis*. A 446 bp DNA fragment amplified with this primer pair was proven to be a partial HDS sequence from *pyropia haitanensis* by Blast analysis. Similar methods and results were observed when cloning *PhHDR*. A 435 bp *PhHDR* DNA fragment amplified with the HDRF/HDRR primer pair was proven to be a partial sequence from HDR from *pyropia haitanensis*. Then, the HDS-5/HDS-3 and HDR-5/HDR-3 primer pairs were designed and used from 5′- and 3′-RACE for *PhHDS and PhHDR*, respectively. Sequence analysis indicated that the full-length cDNA of *PhHDS* was 1801 bp and contained a 192 bp 5′-untranslated region (UTR) and a 153 bp 3′-UTR. The cDNA contained a 1455 bp open reading frame (ORF) and encoded a putative 484 amino acid protein with a molecular weight of 51.60 kDa. The full-length cDNA of *PhHDR* was 1668 bp and contained a 115 bp 5′− untranslated region (UTR) and a 119 bp 3′-UTR. The cDNA contained a 1434 bp open reading frame (ORF) and encoded a putative 477 amino acid protein with a molecular weight of 51.49 kDa. The two cDNA sequences were submitted to GenBank with accession numbers of *PHHDS* (MF101802) and *PHHDR* (MF159558).

### Comparative analysis of *PhHDS* and *PhHDR*

**Figure 2:**
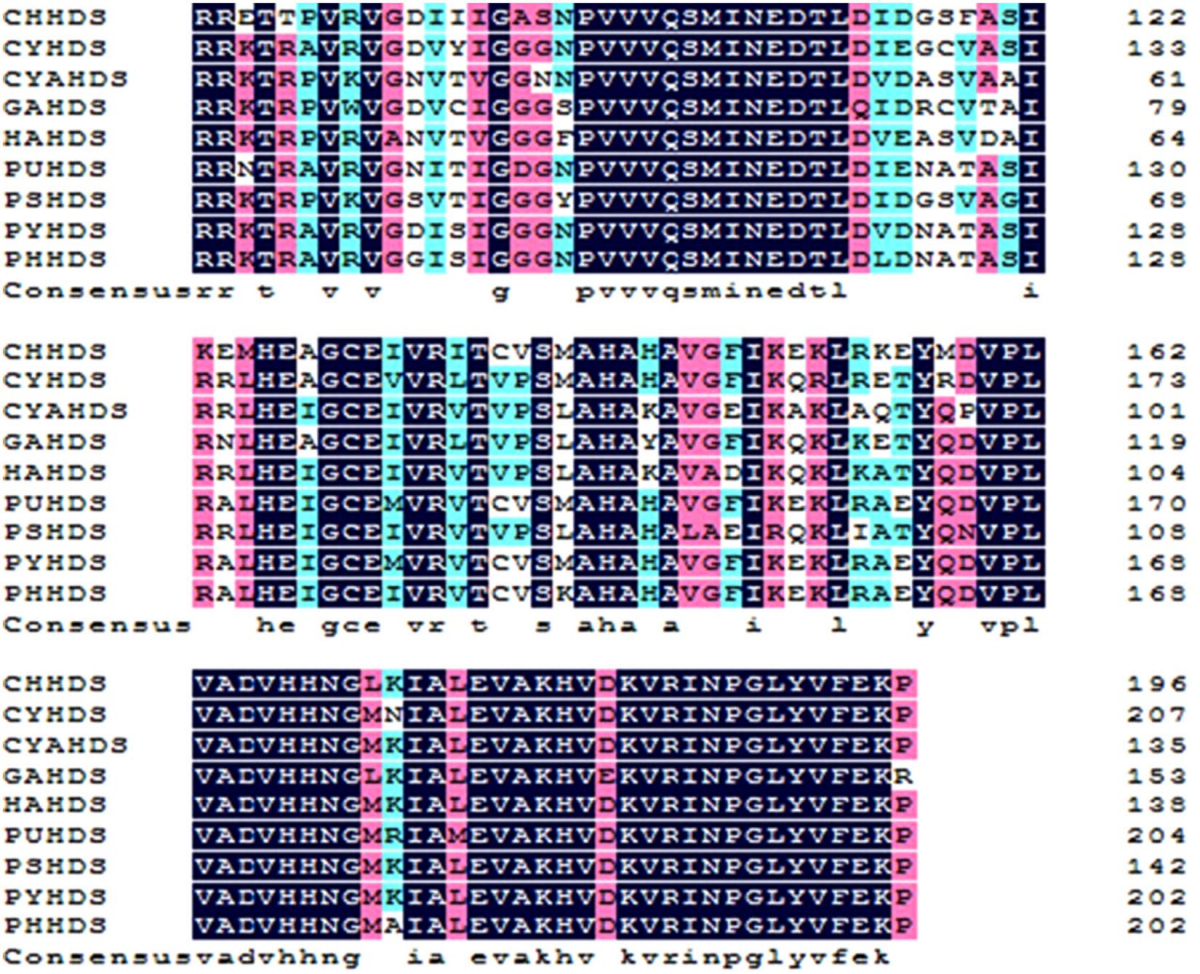
Multiple alignment of the HDS amino acid sequences from different species. Black indicates the amino acids from these species that were the same. Red indicates amino acids where one or two species were different from the other species. Cyan indicates amino acids where three or more species were different from the other species. The aligned HDSs were from *Chondrus crispus* (CHHDS, GenBank accession no.: XP005715044), *Cyanidioschyzon merolae* strain 10D (CYHDS, GenBank accession no.: XP005536899), *Cyanobacterium aponinum* (CYAHDS, GenBank accession no.: WP015219624), *Galdieria sulphuraria* (GAHDS, GenBank accession no.:XP005706298), *Hapalosiphonaceae* (HAHDS, GenBank accession no.: WP026723850), *Porphyra umbilicalis* (PUHDS, GenBank accession no.: OSX72194), *Pseudanabaena sp*. PCC 6802 (PSHDS, GenBank accession no.: WP019498559), *Pyropia yezoensis* (PYHDS, GenBank accession no.: ACI45961), and *Pyropia haitanensis* (PHHDS, GenBank accession no.: ATE45997).

**Figure 3:**
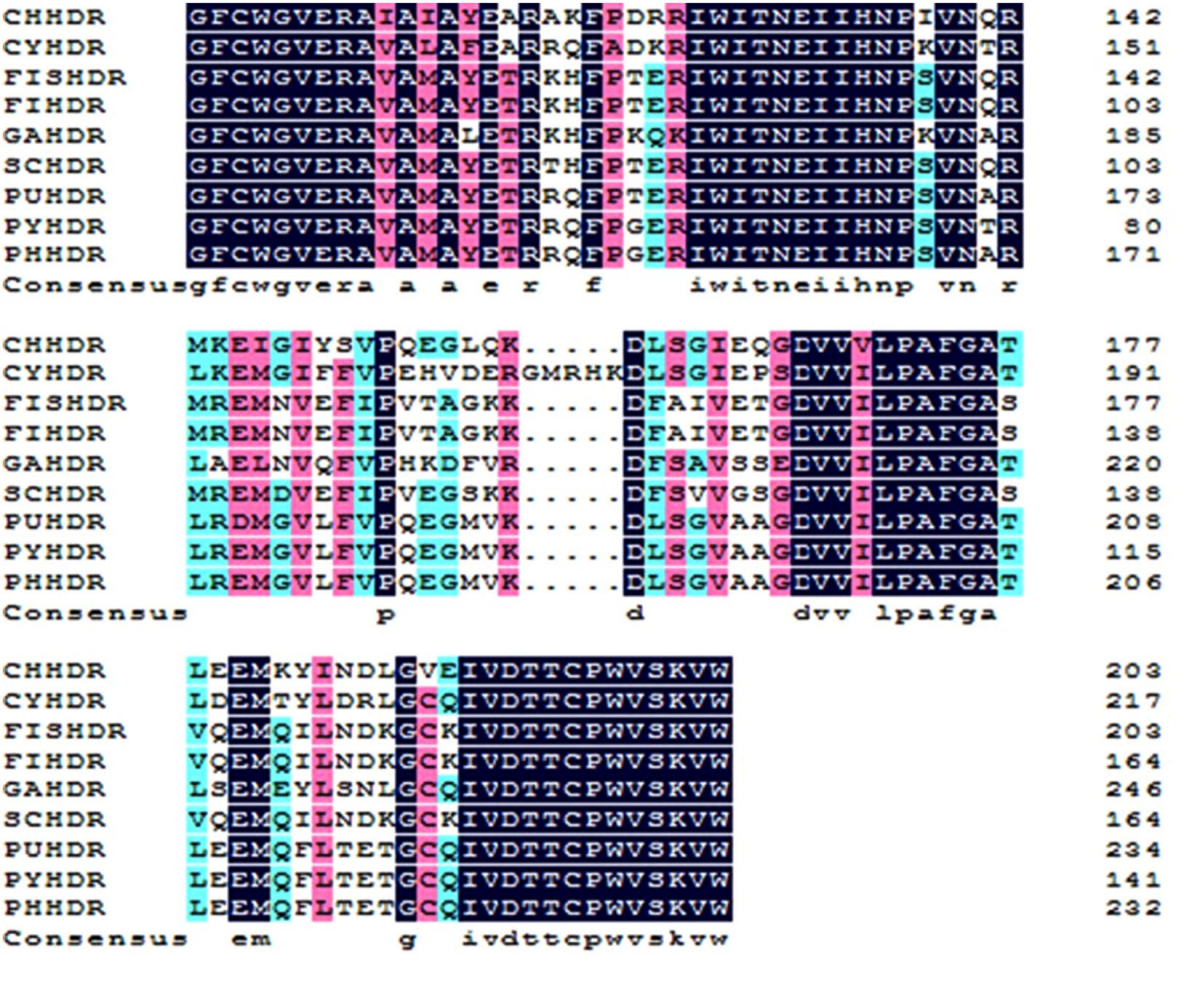
Multiple alignment of HDR amino acid sequences from different species. Black indicates the amino acids from these species were all the same. Red indicates amino acids where one or two species were different from the other species. Cyan indicates amino acids where three or more species were different from the other species. The aligned HDRs were from *Chondrus crispus* (CHHDR, GenBank accession no.: XP 005715383), *Cyanidioschyzon merolae* strain 10D (CYHDR, GenBank accession no.: XP 005534886), *Fischerella sp*. NIES-3754 (FISHDR, GenBank accession no.: BAU08587), *Fischerella* (FIHDR, GenBank accession no.: WP062250827), *Galdieria sulphuraria* (GAHDR, GenBank accession no.: XP005707572), *Scytonema hofmannii* (SCHDR, GenBank accession no.: WP017745471), *Porphyra umbilicalis* (PUHDR, GenBank accession no.: OSX71166), *Pyropia yezoensis* (PYHDR, GenBank accession no.: ACI45962), and *Pyropia haitanensis* (PHHDR, GenBank accession no.: ATE45998).

**Figure 4:**
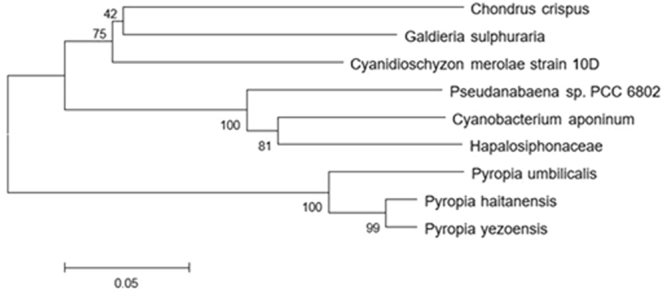
Phylogenetic tree for PHHDS and its related sequences. The tree was constructed with the MEGA programme using the neighbour-joining method. Numbers at nodes represent bootstrap support values after 1000 replicates. The result showed that the PHHDS from *Pyropia haitanensis* which we studied was grouped with Porphyra umbilicalis and *Pyropia yezoensis* as *Pyropia*.

**Figure 5:**
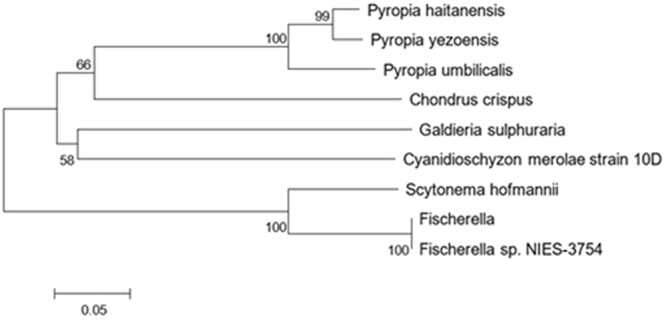
Phylogenetic tree for PHHDR and its related sequences. The tree was constructed with the MEGA programme using the neighbour-joining method. Numbers at nodes represent the bootstrap support values after 1000 replicates. The result showed that the PHHDR from *Pyropia haitanensis* which we studied was grouped with Porphyra umbilicalis and *Pyropia yezoensis* as *Pyropia*.

**Figure 6:**
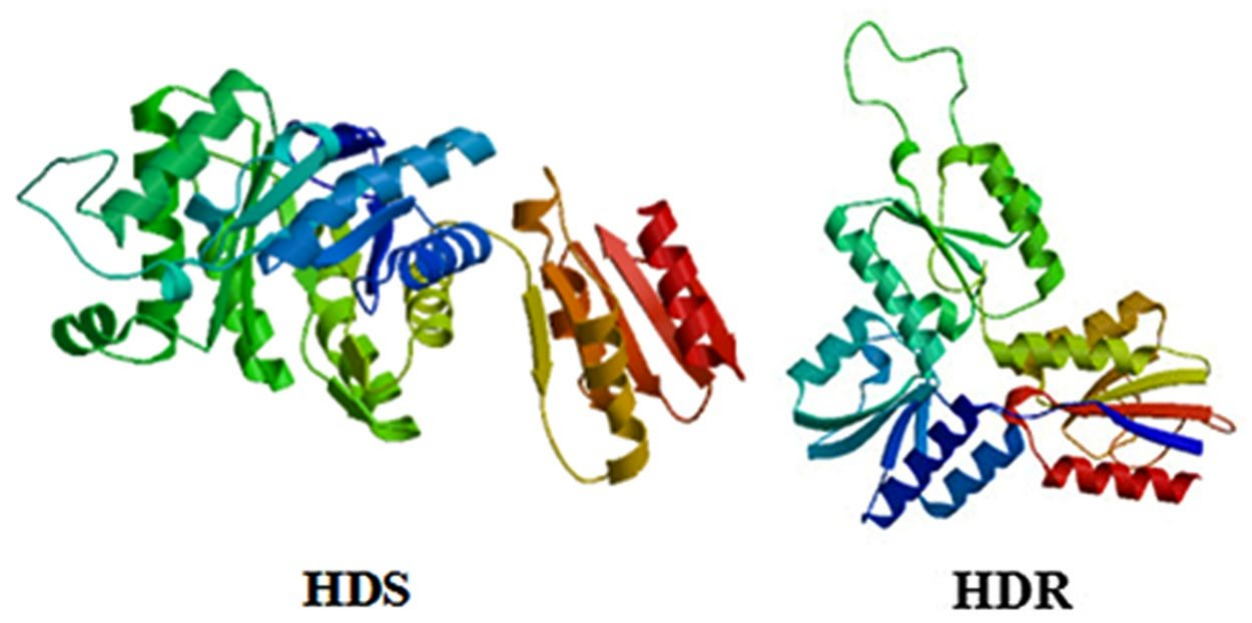
3D structures of the HDS and HDR proteins from *Pyropia haitanensis*. The model was built with SWISS-MODEL and displayed with the Swiss-Pdb Viewer programme. The N-terminal, central catalytic, and C-terminal domains are shown in blue, red, and green, respectively.

We compared the amino acid sequences of *PhHDS and PhHDR* with that of other proteins in GenBank using Protein BLAST (Figs 2 and 3). The results revealed that *PhHDS* shared 95% sequence identity with HDS from *Pyropia yezoensis* (ACI45961.1), 83% similarity with *Porphyra umbilicalis* (OSX72194.1), 64% similarity with *Chondrus crispus* (XP005715044), 72% similarity with *Cyanidioschyzon merolae* strain 10D (XP005536899), 72% similarity with *Cyanobacterium aponinum* (WP015219624), 73% similarity with *Galdieria sulphuraria* (XP005706298), 73% similarity with *Hapalosiphonaceae* (WP026723850) and 72% similarity with *Pseudanabaena sp*. PCC 6802 (WP019498559). Meanwhile, *PhHDR* had 82% homology with HDR from *Porphyra umbilicalis* (OSX71166.1), 96% identity with HDR from *Pyropia yezoensis* (ACI45962.1), 68% identity with *Chondrus crispus* (XP 005715383), 63% identity with *Cyanidioschyzon merolae* strain 10D (XP 005534886), 60% identity with *Fischerella sp*. NIES-3754 (BAU08587), 60% identity with *Fischerella* (WP062250827), 64% identity with *Galdieria sulphuraria* (XP005707572) and 61% identity with *Scytonema hofmannii* (WP017745471). Phylogenetic trees for *PhHDS and PhHDR* were constructed to explore the evolutionary relationship between *PhHDS* and *PhHDR* among the HDSs and HDRs in other algae (Figs 4 and 5), respectively. The 3-D structures of the *PhHDS* and *PhHDR* proteins were predicted by SWISS-MODEL and different 3-D structure models are shown in Fig. 6A and B, respectively. For *PhHDS*, the modelled residue range was 86–470; this structure was based on template 4g9p. The sequence identity for the template was 37.67% and the quality information QMEAN4 was −4.49. For *PhHDR*, modelled residue range was 123–465; this range was based was based on template 4h4c. The sequence identity with the temple was 28.72% and the quality information QMEAN4 was −3.00.

### *PhHDS* and *PhHDR* gene expression profiles in different life histories

To detect *PhHDS and PhHDR* in different life stages, a qPCR approach was used to examine the gene expression patterns between leafy thallus and filamentous conchocelis, which are the two stages in heteromorphic life cycles (see Supplementary Fig. S1). *PhHDS* expression in filamentous conchocelis was 1.81 ± 0.22-fold higher than in leafy thallus, while the expression of *PhHDR* in filamentous conchocelis was 1.98 ± 0.25-fold higher than in leafy thallus. The results suggested that both *PhHDS and PhHDR* expression were approximately 2-fold higher in the conchocelis phase than in the thallus phase.

### *PhHDS* and *PhHDR* gene expression profiles under different growth environments

Leafy thallus was subjected to different environmental factors, and the relative mRNA expression levels of *PhHDS and PhHDR* were investigated using qPCR. The results showed that both *PhHDS and PhHDR* were significantly influenced by temperature (see Supplementary Fig. S2). For *PhHDS*, higher temperatures lower its expression levels. *PhHDS* expression was the highest at 12 °C; this value was 1.96 ± 0.08-fold and 3.04 ± 0.39-fold higher than at 17 °C and 26 °C, respectively. However, the expression levels for *PhHDR* were the opposite, with higher temperature causing higher *PhHDR* expression levels. *PhHDR* expression was the lowest at 12 °C, which was 0.80 ± 0.05-fold and 0.56 ± 0.04-fold than lower than at 17 °C and 26 °C, respectively.

Both *PhHDS and PhHDR* were also influenced by light (see Supplementary Fig. S3). *PhHDS* expression was up-regulated by light; the 120 μmol photons m^−2^·s^−1^ level displayed the highest *PhHDS* expression. This was followed by 240 μmol photons m^−2^·s^−1^. *PhHDS* expression was lowest at 20 μmol photons m^−2^·s^−1^ levels, which was 0.57 ± 0.06-fold and 0.97 ± 0.07-fold less than at 120 μmol photons m^−2^·s^−1^ and 240 μmol photons m^−2^·s^−1^ respectively. Meanwhile, *PhHDR* expression levels were the highest under the light intensity of 120 μmol photons m^−2^·s^−1^, followed by 240 μmol photons m^−2^·s^−1^ and 20 μmol photons m^−2^·s^−1^, which were 0.43 ± 0.03-fold and 0.82 ± 0.05-fold less than 120 μmol photons m^−2^·s^−1^ and 240 μmol photons m^−2^·s^−1^, respectively.

After the treatment with the three different salinities, *PhHDS* expression was highest in medium seawater with 80 salinity, followed by 50 salinity and 30 salinity. *PhHDS* expression at 30 salinity was 0.55 ± 0.01-fold and 0.48 ± 0.05-fold less than at 50 salinity and 80 salinity, respectively. However, the expression levels of *PhHDR* among the three salinities showed few differences. *PhHDR* levels were the highest at 30 salinity, which was 1.07 ± 0.10-fold and 1.27 ± 0.18-fold more than 50 salinity and 80 salinity, respectively. (see Supplementary Fig. S4).

### *PhHDS* and *PhHDR* gene expression due to elicitor treatments

Leafy thallus was placed in sterile distilled water and subjected to methyl jasmonate (MJ) and salicylic acid (SA) treatments to study the expression in response to different elicitors. For MJ treatment, thallus was dipped in either 100 μM or 200 mΜ MJ solutions and placed over a soaked filter paper. Similarly, thallus was dipped in either 1 mM or 2 mM SA solutions for the SA treatment. The control and elicitor-treated samples were harvested after 24 h. Then, the samples were subject to RNA isolation.

The effects of the different MeJA and SA concentrations on *PhHDS* and *PhHDR* expression were examined by qPCR. The data showed that *PhHDS* and *PhHDR* expression levels were up-regulated by MeJA treatment compared to the control group (see Supplementary Fig. S5); the expression levels were higher at the higher concentration. *PhHDS* expression was 1.70 ± 0.22-fold and 5.40 ± 0.46-fold, while *PhHDR* expression was 2.55 ± 0.16-fold and 4.13 ± 0.56-fold compared to the control groups without MeJA treatment. SA treatment also up-regulated the expression levels of the two genes, and their expression was higher at the higher SA concentration. *PhHDS* expression was increased 1.01 ± 0.12-and 4.66 ± 0.29-fold, while *PhHDR* expression was increased 1.21 ± 0.41-fold and 2.45 ± 0.49-fold compared to the control groups without SA treatment (see Supplementary Fig. S6).

## Discussion

Terpenoids are a biological secondary metabolite that directly affect *Pyropia* yield^23^. In terms of their functions, plant terpenoids are divided into the following two categories: primary metabolites and secondary metabolites. The MEP pathway exists in plant plastids, and is one of the major metabolic pathways for the synthesis of secondary metabolites. In this study, we cloned *PhHDS* and *PhHDR*, the last two enzymes in the MEP pathway, from *Pyropia haitanensis* and we analysed the proteins encoded by the two genes using bioinformatics methods. The phylogenetic analysis results showed that both of the genes from *Pyropia haitanensis* were clustered with *Pyropia yezoensis* and *Porphyra umbilicalis*, which meant that the two genes were conserved in *Pyropia*.

Previous research has suggested that metabolic activity was distinctive in different *Pyropia haitanensis* life stages by analysing alternative oxidase (AOX) gene expression^24^. In this study, the expression patterns of *PhHDS* and *PhHDR* during different growth periods showed that *PhHDS* and *PhHDR* expression were approximately 2-fold higher in the conchocelis phase than in the leafy thallus phase. We speculated that there were 2 copies of the corresponding genes because conchocelis is diploid and thallus is haploid; thus, the gene expression differences monitored by qPCR were not considered significant.

Secondary metabolites are the result of biological and non-biological interactions between organisms and the environment during long-term evolution, and secondary metabolites play an important role in organisms in improving survival and coordinating with the environment. Therefore, the production of and changes to secondary metabolites are susceptible to the environment^25,26^. To simulate the complex environments associated with intertidal zones, light, temperature, and salinity were used as environmental factors to study *PhHDS and PhHDR* expression in leafy thallus. The results showed that the expression of the two genes was influenced by the various environments. Transcription levels of the two genes in the MEP pathway obviously increased when Arabidopsis thaliana leaves were irradiated by light^27^. The expression profile of *PuGGPS* in *Porphyra umbilicalis*, which is related to carotenoid biosynthesis, was influenced by light. The gene was more highly expressed under high light conditions^28^. Light is also an influencing factor for the expression of *PhHDS* and *PhHDR*, and their expression levels appeared to increase under higher light intensity. However, *PhHDS* and *PhHDR* was more highly expressed under 120 μmol photons m^−2^·s^−1^ than under 240 μmol photons m^−2^·s^−1^. This finding suggested that excessive light intensity would affect *PhHDS* and *PhHDR* expression. The relationship between the light and temperature dependency of isoprenoid production downstream of the MEP pathway was addressed^29^. The results showed that the metabolic flux of the MEP pathway in spinach increased with light intensity and temperature. At hot temperatures, 2- C-methyl- d -erythritol 2,4-cyclodiphosphate (MEcDP), an intermediate in the MEP pathway in different herbs and trees, accumulated^30^. The results also revealed that MEP pathway metabolic activity was influenced by temperature. In this study, *PhHDS* and *PhHDR* expression levels were diverse at the different temperatures. *PhHDS* was highly expressed at 12 °C. In contrast, *PhHDR* expression was highly expressed at 26 °C. Salinity stress can induce the terpenoid synthesis pathway and synthesize compounds such as monoterpenes and carotenoids^31^. Acetoacetyl-CoA thiolase is a regulatory enzyme in isoprenoid biosynthesis, and real-time RT-PCR analysis indicated that acetoacetyl-CoA thiolase expression levels were highly increased in the roots and leaves under salinity stress^32^. In this study, the expression of *PhHDS* was also influenced by salinity stress. Its expression was increased in both experimental groups compared with the control group; this conclusion was in agreement with the results for terpene synthesis in *Helianthus annuus*, *Saltbushes* and *Disphyma austral* ^33–35^. However, the *PhHDR* expression levels were almost the same threat the different salinities; this phenomenon showed that terpenoid biosynthesis remains the same *Pyropia haitanensis* even under high salinity. The expression levels of the two genes were affected by all three environmental factors, which strongly suggested that the effects of environmental cues on the transcription of the corresponding biosynthetic genes are significant^23^. Environmental stress could induce the emission of algal isoprenoids^36^ and *Pyropia haitanensis* could maintain normal terpenoid metabolic activity even under harsh environments.

MJ and SA have been used intensively either individually or in combination to affect phytoalexin and secondary metabolite synthesis and explore biomolecule biosynthesis^37,38^. MJ and SA can induce the overexpression of genes related to the production of plant secondary metabolites, such as terpenes^39–41^. In this study, *PhHDS* and *PhHDR* expression levels were increased by MJ and SA treatment, and higher concentration of MJ and SA elicited higher gene expression levels, indicating that MJ and SA may increase metabolite production. This conclusion was in agreement with the production of various metabolites in other species, such as sesquiterpene lactone in *Cichorium intybus* ^42^ and artemisinin in *A. annua* ^43^. The major roles of secondary metabolites are to protect organisms from natural enemies and promote survival against biotic and abiotic stresses. Thus, the expression levels of genes associated with terpenoid biosynthesis should increase in *Pyropia haitanensis* upon application of exogenous elicitors.

In conclusion, we first cloned and analysed the *PhHDS* and *PhHDR* genes, which are important for terpene biosynthesis in the MEP pathway in *Pyropia haitanensis*. We also studied their expression profiles under the influence of various environmental factors and through elicitor treatments. Such studies will not only be advantageous for understanding *Pyropia haitanensis* biosynthesis, but will also provide molecular value for studying the MEP pathway in other red algae.

## Methods

### Plant materials

The leafy thallus from *pyropia haitanensis* was collected from Putian, Fujian Province in China (25° 11′ N 119°28′ E) and the conchocelis were purchased from Jiangsu Research Institute of Laver. Before used, the materials were cultivated in the seawater medium, and cool-white fluorescent light was provided in a 12:12 L:D cycle. The cultivation environment for the leafy thallus from *pyropia haitanensis* was as follows: 12 °C, 20 μmol photons m^−2^·s^−1^ and 30 salinity, these conditions were used for the control group in real-time quantitative PCR.

### RNA isolation and cDNA synthesis

Total RNA was isolated from leafy thallus and conchocelis using a MiniBEST Plant RNA Extraction Kit (Takara, Japan) according to the user’s manual. For reverse transcription PCR (Takara Reverse Transcription kit), single-strand cDNA was synthesized from 2 μg of total RNA and stored at −20 °C before used.

### Cloning and sequencing of *PhHDS* and *PhHDR*

All primers used in this study are shown in Table 1. Two pairs of specific primers, HDSF and HDSR and HDRF and HDRR, were designed based on conserved regions from previously known *pyropia yezoensis* sequences (GenBank accession numbers FJ175682.1 and FJ175683.1, respectively) and used to amplify the core cDNA fragments. The reactions were both performed in a total volume of 50 μl containing 39.5 μl ddH2O, 2 μl 10 × PCR Buffer II (Mg2+ plus), 2 μl dNTP Mixture (10 mM), 2 μl cDNA, 1 μl forward primer (20 μM), 1 μl reverse primer (20 μM) and 0.5 μl TaKaRa LA Taq HS (5 U/μl, TaKaRa). The PCR products were cloned into the pEASY-T3 vector (TransGen, China) and sequenced. Rapid amplification of cDNA ends (RACE) PCR was used to obtain the full-length cDNA of *PhHDS* and *PhHDR* according to the manufacturer’s instructions (SMARTer RACE5′/3′ Kit User Manual Extraction Kit (TaKaRa, Japan)). The UPM (Univer Primer Mix) and HDS-5 primer pair was used for the 5′-RACE of *PhHDS*, whereas the HDS-3 and UPM primer pair was used for the 3′-RACE reaction. Meanwhile, the UPM and HDR-5 primer pair was used for the 5′-RACE of *PhHDR*, while the HDR-3 and UPM primer pair was used for the 3′-RACE reaction.

**Table 1:** The primers used in the study.

Gene	Primer name	Sequence (5′-3′)
	HDSF	ATTCCTGTCTGCTACGGCATCCT
	HDSR	GGCAGCGTACCACGTAACTTCTTT
	5′ RACE	
	UPM	CTAATACGACTCACTATAGGGCAAGCAGTGGTATCAACGCAGAGT
**HDS**	HDS-5	CACCCACTCGGTCATTGTGCGTC
	3′ RACE	
	HDS-3	ATCCCCGTCTGCTATGGCATCCTG
	qPCR	
	qHDSF	GAGCCAAACGCAACATCGAA
	qHDSR	AAATCCAGCGTGTCCTCGTT
	HDRF	TACCGAGACGGGGTGCCAGATTG
	HDRR	GCGTCGCAGATGGTGTCATAAGCA
	5′ RACE	
	UPM	CTAATACGACTCACTATAGGGCAAGCAGTGGTATCAACGCAGAGT
**HDR**	HDR-5	GGAACGATAGCGGATGCGGTTCT
	3′ RACE	
	HDR-3	CTGCCCGTGGGTGTCCAAGGTG
	qPCR	
	qHDRF	CGGTGATTCACGGCAAGTGG
	qHDRR	CGCACTCATGGCGTTGGAAA

### Multiple alignment and bioinformatics analysis of *PhHDS* and *PhHDR*

The deduced amino acid sequences of *PhHDS and PhHDR* were aligned with the sequences of other organisms from GenBank using the BLAST programmes (http:// www.ncbi.nlm.nih.gov/BLAST/) and DNAMAN software. The phylogenetic analysis was performed with the MEGA 5.1 software (USA) with the neighbour-joining (NJ) analyses option, and the bootstrap confidence intervals for the phylogenetic tree were based on 1000 replications.

### Different growth environment treatments

To detect the impact of various growth environments on *PhHDS and PhHDR* expression patterns, the following three environmental factors were studied in our research: (1) temperature, (2) light and (3) salinity. For temperature, leafy thallus was subject to three different temperatures (12 °C, 17 °C, and 26 °C) in the same seawater medium for 48 h. For light, leafy thallus was subject to three different light intensities (20 μmol photons m^−2^·s^−1^, 120 μmol photons m^−2^·s^−1^, and 240 μmol photons m^−2^·s^−1^) for 48 h. Leafy thallus was also cultivated in the seawater with three different salinities (30, 50, and 80 salinity) for 2 h. The samples that were subject to different environmental factors were used for RNA isolation in the following experiments.

### Elicitor treatments

Leafy thallus was placed in sterile distilled water and subjected to methyl jasmonate (MJ) and salicylic acid (SA) treatments to study gene expression in response to different elicitors. For MJ treatment, thallus was dipped in either 100 μM or 200 μM MJ solutions and placed over a soaked filter paper. Similarly, thallus was dipped in either 1 mM or 2 mM SA solutions for the SA treatment. The control and elicitor-treated samples were harvested after 24 h, and then the samples were used for RNA isolation.

### Relative quantification by real-time quantitative PCR

The expression patterns of *PhHDS and PhHDR* were explored in different life stages. Additionally, the expression levels of *PhHDS and PhHDR* were also detected under different growth environmental and elicitor treatments. For RT-PCR analysis, a total of 1 μg RNA was used to synthesize cDNA with oligo (dT) primers using the RT-for-PCR kit (Takara, Japan). The expression levels were measured with a Baiyuan ASA-4800 Real Time PCR System using SYBR green fluorescence (TaKaRa) according to the instructions. The following cycling profile was used for amplification: (1) 95 °C for 30 s and (2) 40 cycles of 95 °C for 5 s and 60 °C for 34 s. The relative gene expression was calculated with the 2^−ΔΔCt^ relative quantitative method. The primer sequences used for the amplification of specific genes are listed in Table 1. 18S rRNA primers were designed as an internal control.

### RT-PCR data analysis

All the experiments were repeated at least three times and the data were analysed using the SPSS software (version 22.0 USA). All the data were assessed by one-way analysis of variance (ANOVA) and the significance level was set at P < 0.05. The highest significance level was set at P < 0.01.

## Electronic supplementary material

Supplementary Information
