# Supplementary material for: Molecular cloning and expression analysis of two key genes, HDS and HDR, in the MEP pathway in Pyropia haitanensis
Source: Sci Rep. 2017 Dec 13;7:17499. doi: 10.1038/s41598-017-17521-9 (PMC5727536; doi:10.1038/s41598-017-17521-9)
Supplement: Supplementary file 1 — Supplementary Information [file 41598_2017_17521_MOESM1_ESM.doc]

Molecular cloning and expression analysis of two key genes, HDS and HDR, in the MEP pathway in *Pyropia* *haitanensis*

Yuan He^1^ · Zhihong Yan^2^ · Yu Du^1^ ·Yafeng Ma^1^ ·Songdong Shen^1^*

^1^ Department of cell Biology, School of Biology and Basic Medical, Soochow University, No. 199 Renai Road, Suzhou, China

^2^ Aquaculture technology extending station of Xiuyu District, Putian, China

* Corresponding author, E-mail address: [shensongdong@suda.edu.cn](mailto:shensongdong@suda.edu.cn) Telephone and fax number: 86-513-65880276


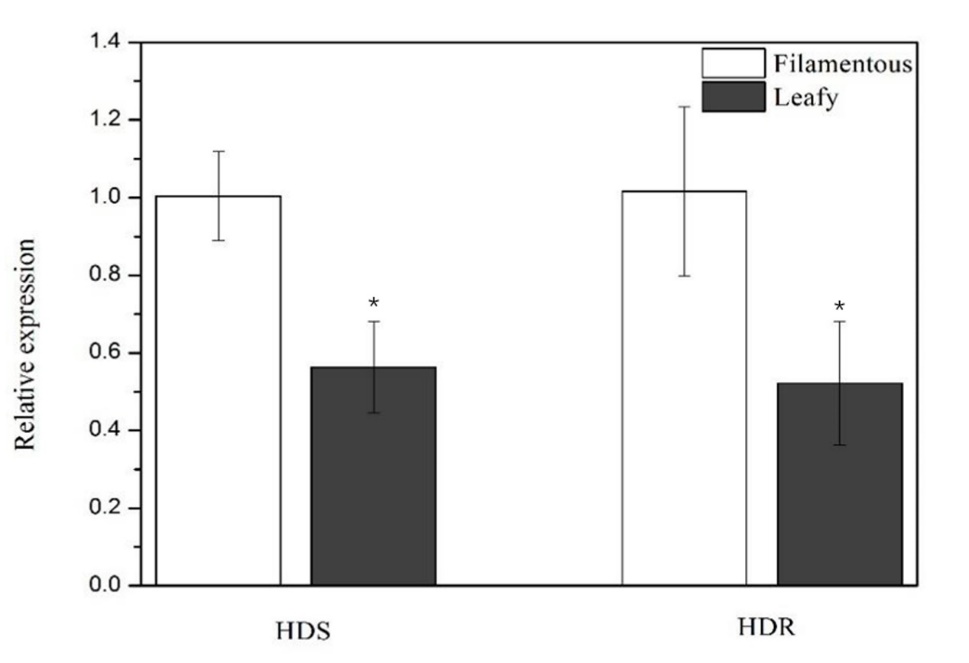


Fig.S1 Shen

Fig. S1. Relative expression of HDS and HDR in different *Pyropia haitanensis* life stages. The results suggested that both *PhHDS and PhHDR* were about 2-fold higher in conchocelis phase than in thallus phase.


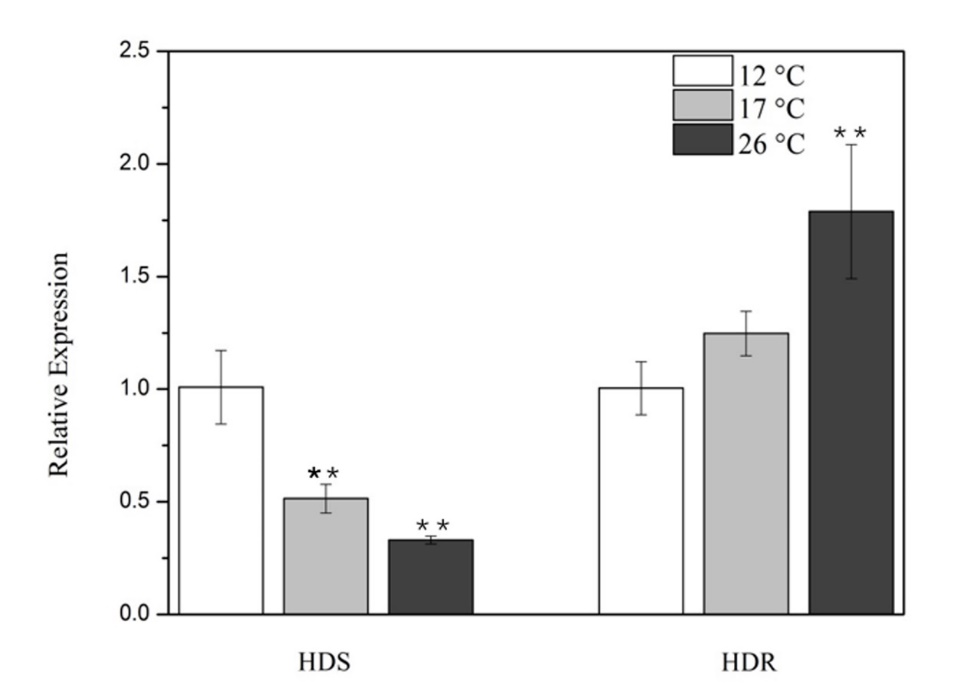


Fig. S2 Shen

Fig. S2. Relative expression of HDS and HDR in *Pyropia haitanensis* under different temperatures. The results showed that both *PhHDS and PhHDR* were siginifcantly influenced by temperature . For *PhHDS*, the higher was the temperature, the lower was the expression level of *PhHDS*. The expression level of *PhHDS* was highest in 12°C. However, the expression level of *PhHDR* was the opposite, the higher was the temperature, the higher was the expression level of *PhHDR*, and the expression levels of *PhHDR* was lowest in 12°C.


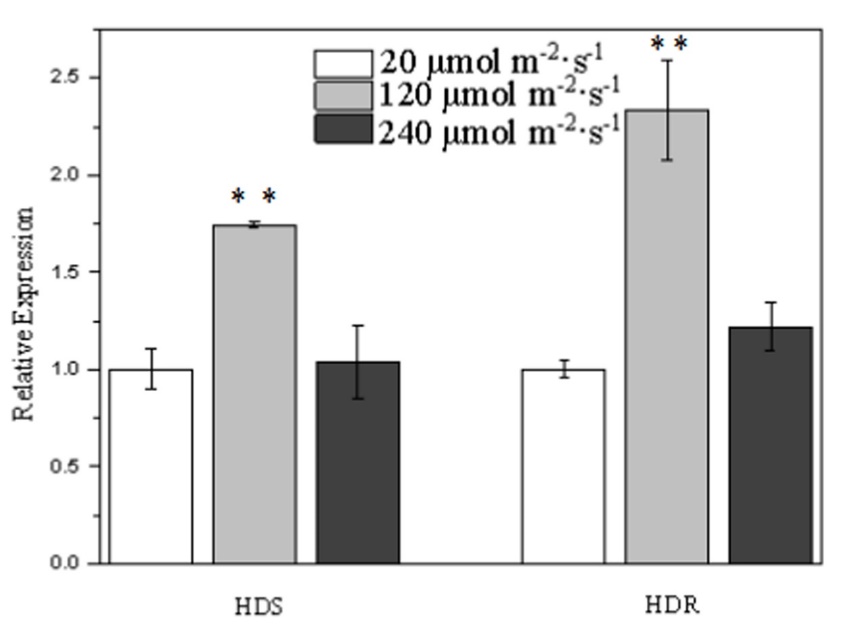


Fig. S3 Shen

Fig. S3. Relative expression of HDS and HDR in *Pyropia haitanensis* under different light intensities. The result showed that both *PhHDS and PhHDR* were also influenced by light. The level of 120 μmol photons m^-2^·s^-1^ was highest, 240 μmol photons m^-2^·s^-1^ was in the next place, and the expression level of *PhHDS* was the lowest when the light intensity was 20 μmol photons m^-2^·s^-1^. The expression levels of *PhHDR* was the highest under the light intensity of 120 μmol photons m^-2^·s^-1^, followed by 240 μmol photons m^-2^·s^-1^, and 20 μmol photons m^-2^·s^-1^ was the lowest.


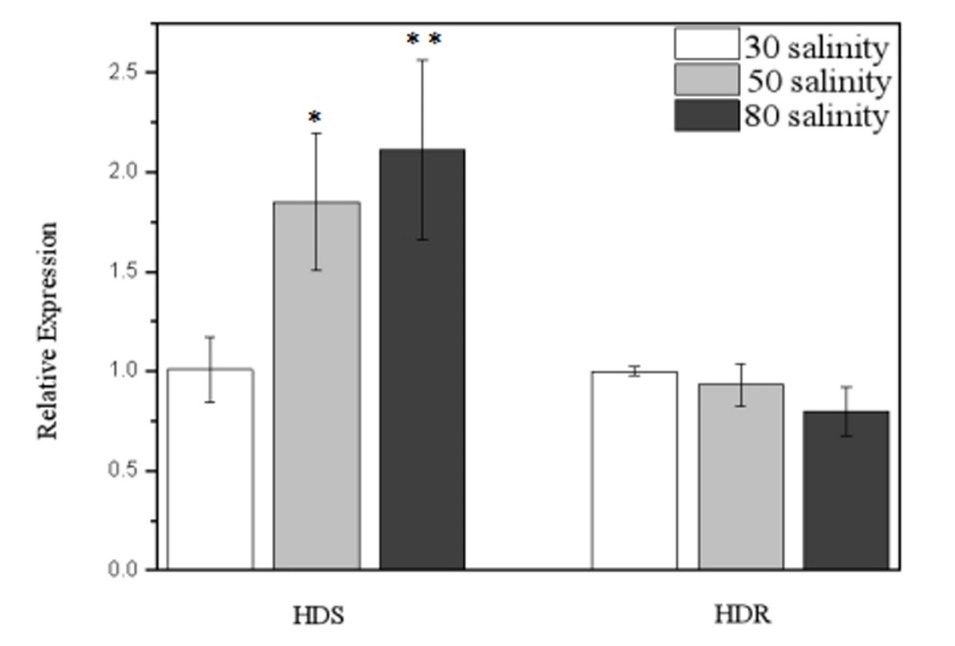


Fig.S4 Shen

Fig. S4. Relative expression of HDS and HDR in *Pyropia haitanensis* under different salinities. The result showed that the expression level of *PhHDS* was highest in seawater medium with 80 salinity, followed by 50 salinity and 30 salinity. However, the expression levels of *PhHDR* among three salinities showed little differences, the level of *PhHDR* was highest in 30 salinity.


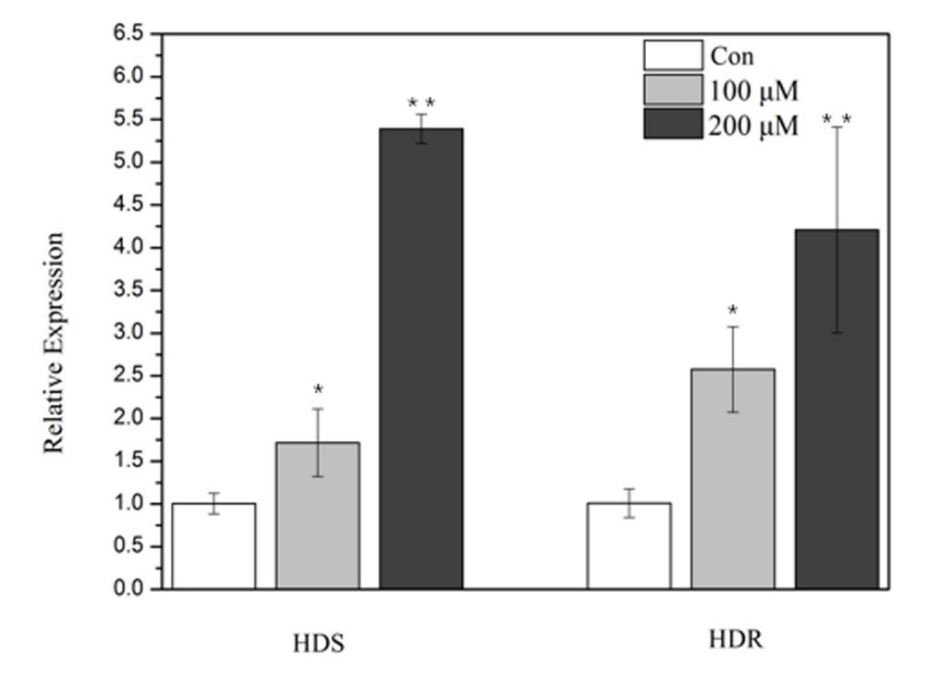


Fig. S5 Shen

Fig. S5. Relative expression of HDS and HDR in *Pyropia haitanensis* due to MeJA treatment. the result showed that MeJA treatment up-regulated the expression levels of *PhHDS* and *PhHDR*, the higher was the concertration of MeJA, the higher was the expression level of *PhHDS* and *PhHDR*.


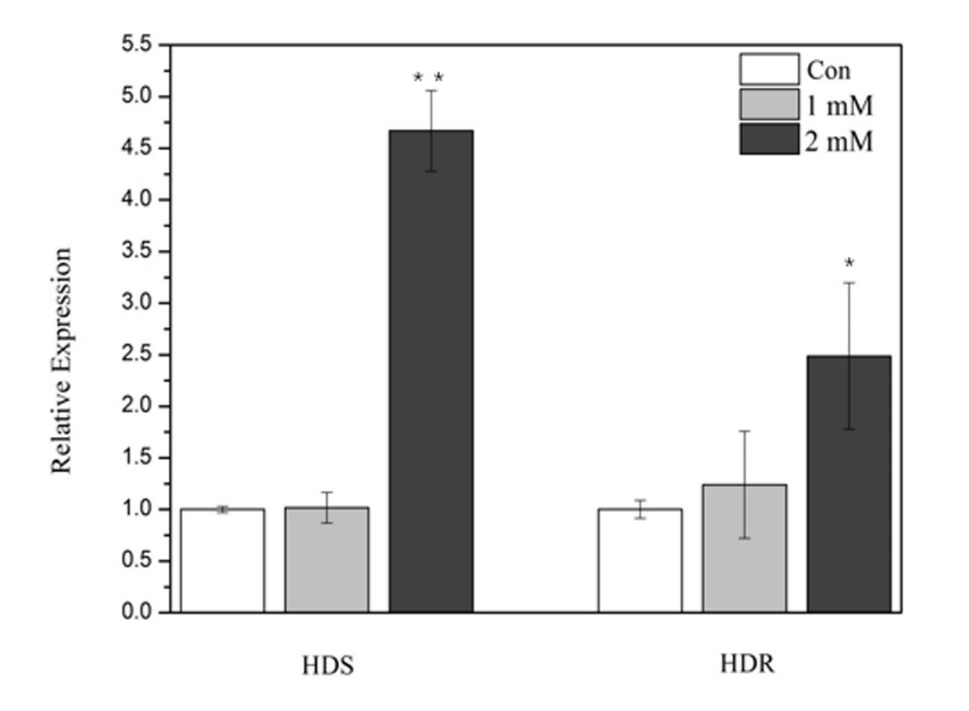


Fig. S6 Shen

Fig. S6. Relative expression of HDS and HDR in *Pyropia haitanensis* due to SA treatment. The result showed that SA treatment up-regulated the expression levels of the two genes and the higher was the concertration of SA, the higher was the expression level of the two genes.
